# (1*S*,3*R*)-3-Ammonio­cyclo­hexa­necarboxyl­ate

**DOI:** 10.1107/S1600536808028080

**Published:** 2008-09-13

**Authors:** Yu Hu, XiaoXia Sun, Ying Guo, Xun Tuo

**Affiliations:** a Experimental Chemistry Center, Nanchang University, Nanchang 330031, People’s Republic of China; b Jiangxi Key Laboratory of Organic Chemistry, Jiangxi Science and Technology Normal University, Nanchang 330013, People’s Republic of China

## Abstract

The title γ-amino­butyric acid, C_7_H_13_NO_2_, exists as a zwitterion. The crystal structure is stabilized by a network of inter­molecular N—H⋯O hydrogen bonds, forming a two-dimensional bilayer. An inter­molecular C—H⋯O hydrogen bond is also observed.

## Related literature

For related literature, see: Allan *et al.* (1981 ▶); Ávila *et al.* (2004 ▶); Fábián *et al.* (2005 ▶); Granja (2004 ▶); Hu *et al.* (2006 ▶); Schousboe (2000 ▶).
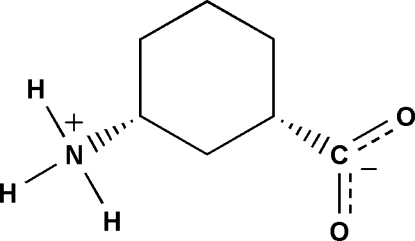


## Experimental

### 

#### Crystal data

C_7_H_13_NO_2_
*M* *_r_* = 143.18
Orthorhombic, 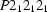

*a* = 5.5130 (10) Å
*b* = 6.1282 (9) Å
*c* = 22.518 (4) Å
*V* = 760.8 (2) Å^3^
*Z* = 4
Mo *K*α radiation
μ = 0.09 mm^−1^
*T* = 293 (2) K
0.48 × 0.38 × 0.30 mm

#### Data collection

Bruker SMART 1K area-detector diffractometer
Absorption correction: multi-scan (*SADABS*; Sheldrick, 1996 ▶) *T*_min_ = 0.958, *T*_max_ = 0.973
1150 measured reflections
1107 independent reflections
891 reflections with *I* > 2σ(*I*)
*R*_int_ = 0.013

#### Refinement

*R*[*F* ^2^ > 2σ(*F* ^2^)] = 0.038
*wR*(*F* ^2^) = 0.093
*S* = 0.97
1107 reflections
92 parameters
H-atom parameters constrained
Δρ_max_ = 0.15 e Å^−3^
Δρ_min_ = −0.20 e Å^−3^

### 

Data collection: *SMART* (Bruker, 1999 ▶); cell refinement: *SAINT-Plus* (Bruker, 1999 ▶); data reduction: *SAINT-Plus*; program(s) used to solve structure: *SHELXS97* (Sheldrick, 2008 ▶); program(s) used to refine structure: *SHELXL97* (Sheldrick, 2008 ▶); molecular graphics: *SHELXTL* (Sheldrick, 2008 ▶); software used to prepare material for publication: *SHELXTL*.

## Supplementary Material

Crystal structure: contains datablocks I, global. DOI: 10.1107/S1600536808028080/wn2278sup1.cif

Structure factors: contains datablocks I. DOI: 10.1107/S1600536808028080/wn2278Isup2.hkl

Additional supplementary materials:  crystallographic information; 3D view; checkCIF report

## Figures and Tables

**Table 1:** Hydrogen-bond geometry (Å, °)

*D*—H⋯*A*	*D*—H	H⋯*A*	*D*⋯*A*	*D*—H⋯*A*
N1—H1*C*⋯O1^i^	0.89	1.84	2.725 (2)	172
N1—H1*D*⋯O2^ii^	0.89	2.00	2.849 (2)	160
N1—H1*E*⋯O1^iii^	0.89	1.89	2.772 (2)	170
C6—H6⋯O2^iv^	0.98	2.55	3.472 (2)	156

Symmetry codes: (i) 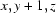; (ii) 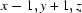; (iii) 
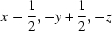
; (iv) 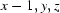.

**Fig. 1.**
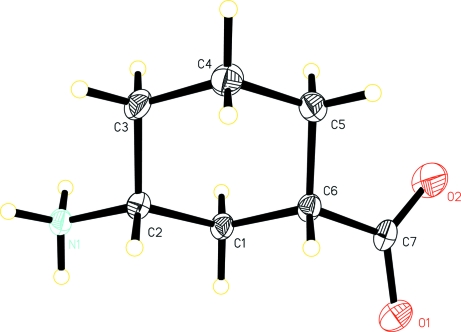
A view of the molecular structure of the title compound, with anisotropic displacement parameters drawn at the 50% probability level. H atoms are represented by spheres of arbitrary radius.

**Fig. 2.**
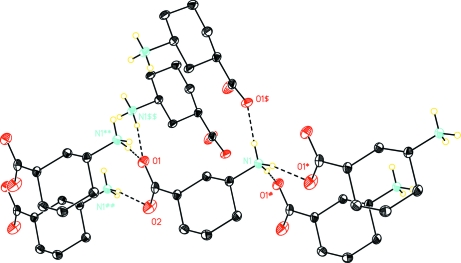
A view of the hydrogen-bonded molecular strands (dashed lines). The strands are aligned parallel to the crystallographic b axis. H atoms not involved in hydrogen bonding have been omitted for clarity. Symmetry codes: (*) x-1,1 + y,z; (**) x,y-1,z; (#)x,1 + y,z; (##) 2 + x,y-1,z; ($)x-1/2,1/2 - y,-z; ($$) 1/2 + x,1/2 - y,-z

**Fig. 3.**
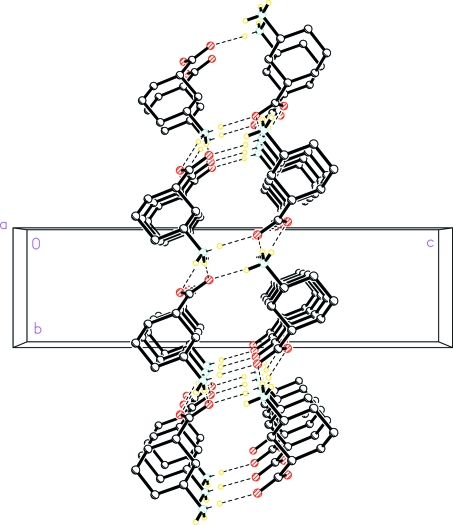
A crystal packing diagram, viewed down the a axis, showing the layer architecture.

## supplementary crystallographic information

### Comment

The importance of the inhibitory neurotransmitter, γ-aminobutyric acid (GABA), in certain neurological and psychiatric disorders has become generally accepted (Schousboe *et al.*, 2000). As an analogue of GABA, 3-aminocyclohexanecarboxylic acid has been investigated in structure–activity studies of conformationally restricted analogues (Allan *et al.*, 1981). From another point of view, self-assembling peptide nanotubes, which contain 3-aminocyclohexanecarboxylic acid, have structural and functional properties that may be suitable for various applications in biology and material science (Granja, 2004). The structure of *1S*,*3R*-3-aminocyclohexanecarboxylic acid was elucidated by spectroscopic analysis. Here we report its crystal structure.

The X-ray crystallographic study confirms the molecular structure previously proposed on the basis of spectroscopic data. The title compound exists as a zwitterion, containing an ammonium group and a carboxylate group (Fig. 1) and amino acid units are linked, in a head-to-tail fashion, by hydrogen bonds (Fig. 2 and Table 1); this is very often observed in the crystal structures of amino acids (Ávila *et al.*, 2004; Fábián *et al.*, 2005). The hydrogen bonds result in a two-dimensional bilayer structure parallel to the *bc* plane (Fig. 3).

### Experimental

*1S*,*3R*-3-amino-cyclohexanecarboxylic acid was synthesized and resolved from 3-cyclohexenecarboxylic acid (Hu *et al.*, 2006). Its identity was confirmed by NMR and HRMS. ^1^H NMR in D_2_O (300 MHz): 3.19–3.26 (m, 1H), 2.16–2.28 (m, 2H), 1.89–2.03 (m, 3H), 1.27–1.50 (m, 4H). ^13^C NMR in D_2_O (75 MHz): 183.96, 49.91, 45.02, 33.55, 29.89, 28.48, 23.30 HRMS calcd for C_7_H_12_NO_2_ 142.0863, found 142.0859. Single crystals suitable for X-ray diffraction analysis were obtained by the slow diffusion of acetone into an aqueous solution of the title compound.

### Refinement

Carbon-bound H atoms were positioned geometrically and were treated as riding on their parent atoms, with C—H distances in the range 0.97–0.98 Å, with *U*_iso_(H) = 1.2 times *U*_eq_ of the parent atom. H atoms attached to N1 were located in difference Fourier maps and refined initially with distance restraints of 0.89 Å. They were then repositioned geometrically and refined as riding, with N—H = 0.89 Å and with *U*_iso_(H) = 1.5 times *U*_eq_(N). In the absence of significant anomalous scattering effects, Friedel pairs were merged.

### Crystal data

**Table:**

C_7_H_13_NO_2_	*F*(000) = 312
*M**_r_* = 143.18	*D*_x_ = 1.250 Mg m^−^^3^
Orthorhombic, *P*2_1_2_1_2_1_	Mo *K*α radiation, λ = 0.71073 Å
Hall symbol: P 2ac 2ab	Cell parameters from 31 reflections
*a* = 5.513 (1) Å	θ = 4.9–13.6°
*b* = 6.1282 (9) Å	µ = 0.09 mm^−^^1^
*c* = 22.518 (4) Å	*T* = 293 K
*V* = 760.8 (2) Å^3^	Block, colourless
*Z* = 4	0.48 × 0.38 × 0.30 mm

### Data collection

**Table:**

Bruker SMART 1K area-detector diffractometer	1107 independent reflections
Radiation source: fine-focus sealed tube	891 reflections with *I* > 2σ(*I*)
graphite	*R*_int_ = 0.013
φ and ω scans	θ_max_ = 28.0°, θ_min_ = 1.8°
Absorption correction: multi-scan (SADABS; Sheldrick, 1996)	*h* = 0→7
*T*_min_ = 0.958, *T*_max_ = 0.973	*k* = 0→8
1150 measured reflections	*l* = −1→29

### Refinement

**Table:**

Refinement on *F*^2^	Primary atom site location: structure-invariant direct methods
Least-squares matrix: full	Secondary atom site location: difference Fourier map
*R*[*F*^2^ > 2σ(*F*^2^)] = 0.038	Hydrogen site location: inferred from neighbouring sites
*wR*(*F*^2^) = 0.093	H-atom parameters constrained
*S* = 0.97	*w* = 1/[σ^2^(*F*_o_^2^) + (0.057*P*)^2^] where *P* = (*F*_o_^2^ + 2*F*_c_^2^)/3
1107 reflections	(Δ/σ)_max_ < 0.001
92 parameters	Δρ_max_ = 0.15 e Å^−^^3^
0 restraints	Δρ_min_ = −0.20 e Å^−^^3^

### Special details

**Table:**

Geometry. All e.s.d.'s (except the e.s.d. in the dihedral angle between two l.s. planes) are estimated using the full covariance matrix. The cell e.s.d.'s are taken into account individually in the estimation of e.s.d.'s in distances, angles and torsion angles; correlations between e.s.d.'s in cell parameters are only used when they are defined by crystal symmetry. An approximate (isotropic) treatment of cell e.s.d.'s is used for estimating e.s.d.'s involving l.s. planes.
Refinement. Refinement of *F*^2^ against ALL reflections. The weighted *R*-factor *wR* and goodness of fit *S* are based on *F*^2^, conventional *R*-factors *R* are based on *F*, with *F* set to zero for negative *F*^2^. The threshold expression of *F*^2^ > σ(*F*^2^) is used only for calculating *R*-factors(gt) *etc*. and is not relevant to the choice of reflections for refinement. *R*-factors based on *F*^2^ are statistically about twice as large as those based on *F*, and *R*- factors based on ALL data will be even larger.

### Fractional atomic coordinates and isotropic or equivalent isotropic displacement parameters (Å^2^)

**Table:**

	*x*	*y*	*z*	*U*_iso_*/*U*_eq_	
C1	0.2478 (4)	0.3817 (3)	0.08035 (7)	0.0282 (4)	
H1A	0.3925	0.4696	0.0750	0.034*	
H1B	0.2011	0.3237	0.0419	0.034*	
C2	0.0440 (3)	0.5232 (3)	0.10484 (8)	0.0262 (4)	
H2	−0.1034	0.4346	0.1077	0.031*	
C3	0.1066 (4)	0.6102 (3)	0.16649 (8)	0.0339 (5)	
H3A	0.2466	0.7055	0.1639	0.041*	
H3B	−0.0285	0.6946	0.1818	0.041*	
C4	0.1614 (4)	0.4219 (3)	0.20854 (8)	0.0369 (5)	
H4A	0.2069	0.4796	0.2471	0.044*	
H4B	0.0166	0.3340	0.2137	0.044*	
C5	0.3656 (4)	0.2795 (3)	0.18478 (8)	0.0347 (5)	
H5A	0.3926	0.1580	0.2116	0.042*	
H5B	0.5141	0.3641	0.1827	0.042*	
C6	0.3023 (3)	0.1930 (3)	0.12313 (7)	0.0265 (4)	
H6	0.1526	0.1080	0.1271	0.032*	
C7	0.4929 (3)	0.0418 (3)	0.09604 (9)	0.0303 (4)	
N1	−0.0035 (3)	0.7099 (2)	0.06373 (6)	0.0293 (4)	
H1C	0.1290	0.7923	0.0610	0.044*	
H1D	−0.1256	0.7897	0.0778	0.044*	
H1E	−0.0422	0.6588	0.0280	0.044*	
O1	0.4251 (3)	−0.0725 (2)	0.05224 (6)	0.0405 (4)	
O2	0.6983 (3)	0.0343 (3)	0.11737 (8)	0.0611 (6)	

### Atomic displacement parameters (Å^2^)

**Table:**

	*U*^11^	*U*^22^	*U*^33^	*U*^12^	*U*^13^	*U*^23^
C1	0.0320 (10)	0.0277 (9)	0.0249 (8)	0.0087 (9)	0.0012 (7)	−0.0031 (7)
C2	0.0268 (9)	0.0234 (8)	0.0285 (8)	0.0039 (8)	0.0018 (8)	−0.0017 (7)
C3	0.0454 (12)	0.0292 (9)	0.0271 (9)	0.0112 (10)	0.0044 (9)	−0.0050 (8)
C4	0.0503 (12)	0.0357 (10)	0.0247 (8)	0.0060 (11)	0.0034 (9)	−0.0002 (9)
C5	0.0402 (11)	0.0347 (10)	0.0291 (9)	0.0074 (10)	−0.0035 (9)	0.0004 (9)
C6	0.0239 (9)	0.0228 (8)	0.0329 (9)	0.0036 (8)	0.0014 (8)	−0.0014 (8)
C7	0.0300 (9)	0.0212 (8)	0.0397 (10)	0.0031 (9)	0.0059 (9)	0.0012 (9)
N1	0.0322 (8)	0.0287 (7)	0.0271 (7)	0.0101 (8)	−0.0014 (7)	−0.0030 (7)
O1	0.0442 (8)	0.0393 (8)	0.0379 (8)	0.0026 (8)	0.0110 (6)	−0.0114 (7)
O2	0.0320 (8)	0.0617 (11)	0.0896 (13)	0.0192 (9)	−0.0095 (8)	−0.0294 (11)

### Geometric parameters (Å, °)

**Table:**

C1—C2	1.523 (2)	C4—H4B	0.9700
C1—C6	1.535 (2)	C5—C6	1.526 (2)
C1—H1A	0.9700	C5—H5A	0.9700
C1—H1B	0.9700	C5—H5B	0.9700
C2—N1	1.495 (2)	C6—C7	1.528 (2)
C2—C3	1.527 (2)	C6—H6	0.9800
C2—H2	0.9800	C7—O2	1.231 (2)
C3—C4	1.523 (3)	C7—O1	1.266 (2)
C3—H3A	0.9700	N1—H1C	0.8900
C3—H3B	0.9700	N1—H1D	0.8900
C4—C5	1.522 (3)	N1—H1E	0.8900
C4—H4A	0.9700		
			
C2—C1—C6	110.26 (14)	H4A—C4—H4B	108.0
C2—C1—H1A	109.6	C4—C5—C6	110.48 (16)
C6—C1—H1A	109.6	C4—C5—H5A	109.6
C2—C1—H1B	109.6	C6—C5—H5A	109.6
C6—C1—H1B	109.6	C4—C5—H5B	109.6
H1A—C1—H1B	108.1	C6—C5—H5B	109.6
N1—C2—C1	109.94 (14)	H5A—C5—H5B	108.1
N1—C2—C3	109.61 (14)	C5—C6—C7	114.62 (15)
C1—C2—C3	111.20 (15)	C5—C6—C1	110.72 (15)
N1—C2—H2	108.7	C7—C6—C1	109.93 (14)
C1—C2—H2	108.7	C5—C6—H6	107.1
C3—C2—H2	108.7	C7—C6—H6	107.1
C4—C3—C2	110.22 (15)	C1—C6—H6	107.1
C4—C3—H3A	109.6	O2—C7—O1	123.73 (19)
C2—C3—H3A	109.6	O2—C7—C6	119.95 (18)
C4—C3—H3B	109.6	O1—C7—C6	116.32 (17)
C2—C3—H3B	109.6	C2—N1—H1C	109.5
H3A—C3—H3B	108.1	C2—N1—H1D	109.5
C5—C4—C3	111.27 (15)	H1C—N1—H1D	109.5
C5—C4—H4A	109.4	C2—N1—H1E	109.5
C3—C4—H4A	109.4	H1C—N1—H1E	109.5
C5—C4—H4B	109.4	H1D—N1—H1E	109.5
C3—C4—H4B	109.4		

### Hydrogen-bond geometry (Å, °)

**Table:**

*D*—H···*A*	*D*—H	H···*A*	*D*···*A*	*D*—H···*A*
N1—H1C···O1^i^	0.89	1.84	2.725 (2)	172.
N1—H1D···O2^ii^	0.89	2.00	2.849 (2)	160.
N1—H1E···O1^iii^	0.89	1.89	2.772 (2)	170.
C6—H6···O2^iv^	0.98	2.55	3.472 (2)	156.

Symmetry codes: (i) *x*, *y*+1, *z*; (ii) *x*−1, *y*+1, *z*; (iii) *x*−1/2, −*y*+1/2, −*z*; (iv) *x*−1, *y*, *z*.

## References

[bb1] Allan, R. D., Johnston, G. A. R. & Twitchin, B. (1981). *Aust. J. Chem.* **34**, 2231–2236.

[bb2] Ávila, E. E., Mora, A. J., Delgado, G. E., Ramírez, B. M., Bahsas, A. & Koteich, S. (2004). *Acta Cryst.* C**60**, o759–o761. 10.1107/S0108270104020943 15467151

[bb3] Bruker (1999). *SMART* and *SAINT-Plus* Bruker AXS Inc, Madison, Wisconsin, USA.

[bb4] Fábián, L., Kálmán, A., Argay, G., Bernáth, G. & Gyarmati, Z. Cs. (2005). *Cryst. Growth Des.* **5**, 773–782.

[bb5] Granja, J. R. (2004). Intl Patent WO 2 004 052 916.

[bb6] Hu, Y., Yu, S. L., Yang, Y. J., Zhu, J. & Deng, J. G. (2006). *Chin. J. Chem.* **24**, 795–799.

[bb7] Schousboe, A. (2000). *Neurochem. Res.* **25**, 1241–1244. 10.1023/a:1007692012048 11059798

[bb8] Sheldrick, G. M. (1996). *SADABS* University of Göttingen, Germany.

[bb9] Sheldrick, G. M. (2008). *Acta Cryst.* A**64**, 112–122. 10.1107/S0108767307043930 18156677

